# Uncommon presentation of potential medication-related osteonecrosis of the jaw

**DOI:** 10.1186/s40064-016-1902-5

**Published:** 2016-02-27

**Authors:** Seong-Gon Kim, HaeYong Kweon, Suk-Keun Lee

**Affiliations:** Department of Oral and Maxillofacial Surgery, College of Dentistry, Gangneung-Wonju National University, Gangneung, Gangwondo 210-702 Republic of Korea; Sericultural and Apicultural Materials Division, National Academy of Agricultural Science, Jeonju, Republic of Korea; Department of Oral Pathology, College of Dentistry, Gangneung-Wonju National University, Gangneung, Republic of Korea

**Keywords:** Medication-related osteonecrosis of the jaw, Anti-angiogenic drug, Macrophage, Inflammation

## Abstract

**Background:**

This article presents a patient with potential atypical medication-related osteonecrosis of the jaw and reviews related literatures.

**Case presentation:**

A 52-year-old male showed pain in the left buccal area and had numbness on the left lower lip area. He received medications having anti-angiogenic effect for 4 years. He did not receive irradiation of the jaw regions. In histological view, most of the adipocytes were destroyed and disappeared in the scanty vascular marrow tissue, resulting in the replacement of the fatty necrosis with variable sized vacuolated empty spaces. In the immunohistochemistry analysis, the infiltrated macrophages into the marrow stromal tissue were strongly positive for lysozymes. These findings demonstrate that the presented osteonecrosis underwent a chronic and persistent granulomatous inflammatory reaction.

**Conclusions:**

We conclude that the present case might have been caused by anti-angiogenic drug abuse, affecting the reduction of the mandibular marrow vascularity and subsequently inducing fatty necrosis and an extensive osteolytic change of the mandible.

## Background

Since bisphosphonate-related osteonecrosis of the jaw bone was first reported, many reports regarding medication-related osteonecrosis of the jaw (MRONJ) have followed. Most cases are related to dental injury or teeth in their onset or pathogenesis, and jaw bone exposure over 8 weeks is a prerequisite for diagnosis (Khosla et al. 2007; Ruggiero et al. 2009). Additionally, MRONJ patients have a positive history for anti-angiogenic drugs, but no history of irradiation (Ruggiero et al. 2009). Recently, we observed a case of osteonecrosis of the jaw bone. Unusually, the lesion is located below the inferior alveolar canal and is not related to a dental lesion. The patient had been prescribed medicine for over 3 years for the symptomatic treatment of pneumoconiosis. The potential cause for osteonecrosis is analyzed in this publication.

## Case report

**Fig. 1:**
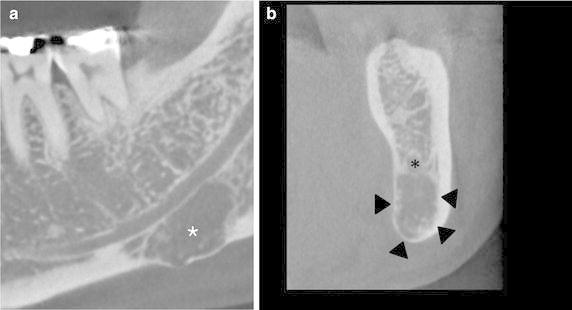
Computerized tomogram findings. **a** The osteolytic lesion was localized in the left first and second molar area below the inferior alveolar canal. **b** The lingual cortex adjacent to the lesion showed erosive changes

On Apr 16, 2015 a 52-year-old male was referred from a local clinic because of a radiolucent lesion in the left mandibular angle area. The patient showed pain in the left buccal area and had numbness on the left lower lip area. He received an operation under general anesthesia because of pneumothorax in 1998. He had been diagnosed with pneumoconiosis in 2001. Because the symptoms of pneumoconiosis were aggravated from 2011, he received medications, such as ozagrel, erdostein, acebrophyline, and Synatura^®^ (Ahngook Pharm, Seoul, Korea), intermittently. Synatura^®^ is an herbal medicine mixed with ivy leaf and coptis stalk. From February 2015, he received olmesartan (an angiotensin receptor antagonist) for the treatment of hypertension. From March 2015, he received atrovastatin calcium for the treatment of hyperlipidemia. He did not receive irradiation of the jaw regions. From a computerized tomogram examination, we observed that the lesion was localized in the left first and second molar area below the inferior alveolar canal (Fig. 1a). The lingual cortex adjacent to the lesion showed an erosive change (Fig. 1b). Based on the radiologic findings, the lesion was primarily diagnosed as Stafne’s bone cyst.

**Fig. 2:**
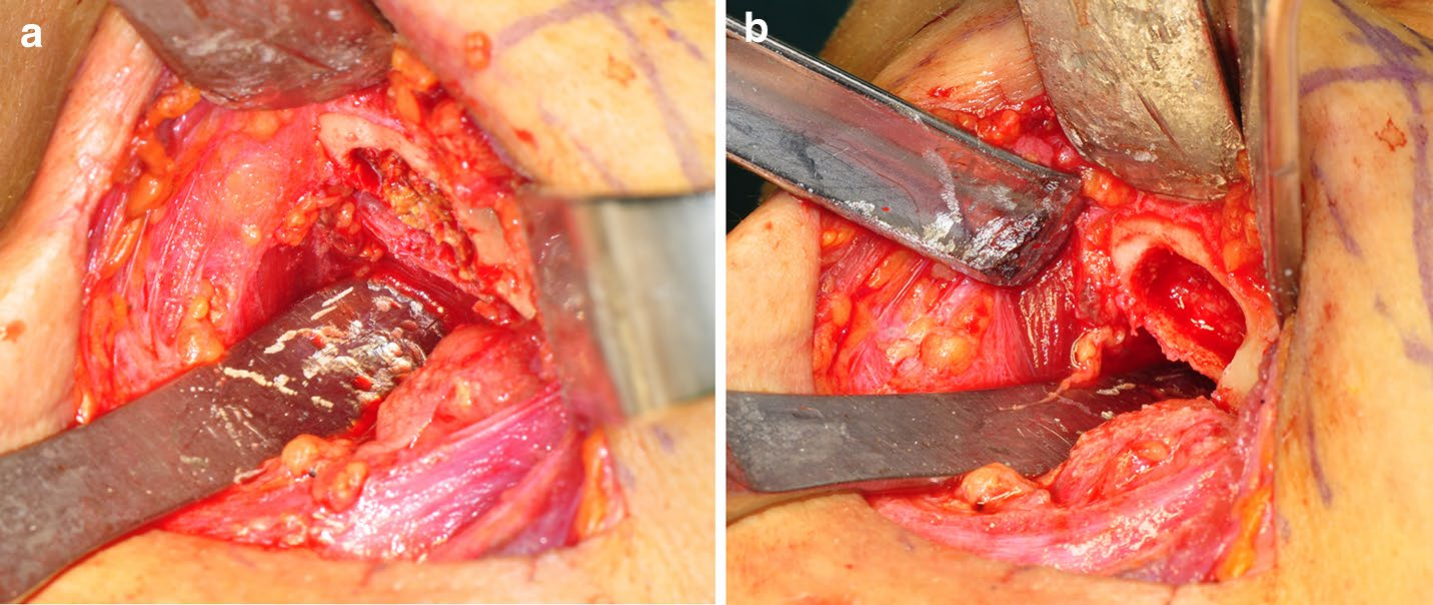
Intra-operative views. **a** The *yellow colored* semi-solid materials were filled with a bone marrow cavity. **b** After removing the osteolytic lesion, the surrounding cortical bone was thin but appeared intact

**Fig. 3:**
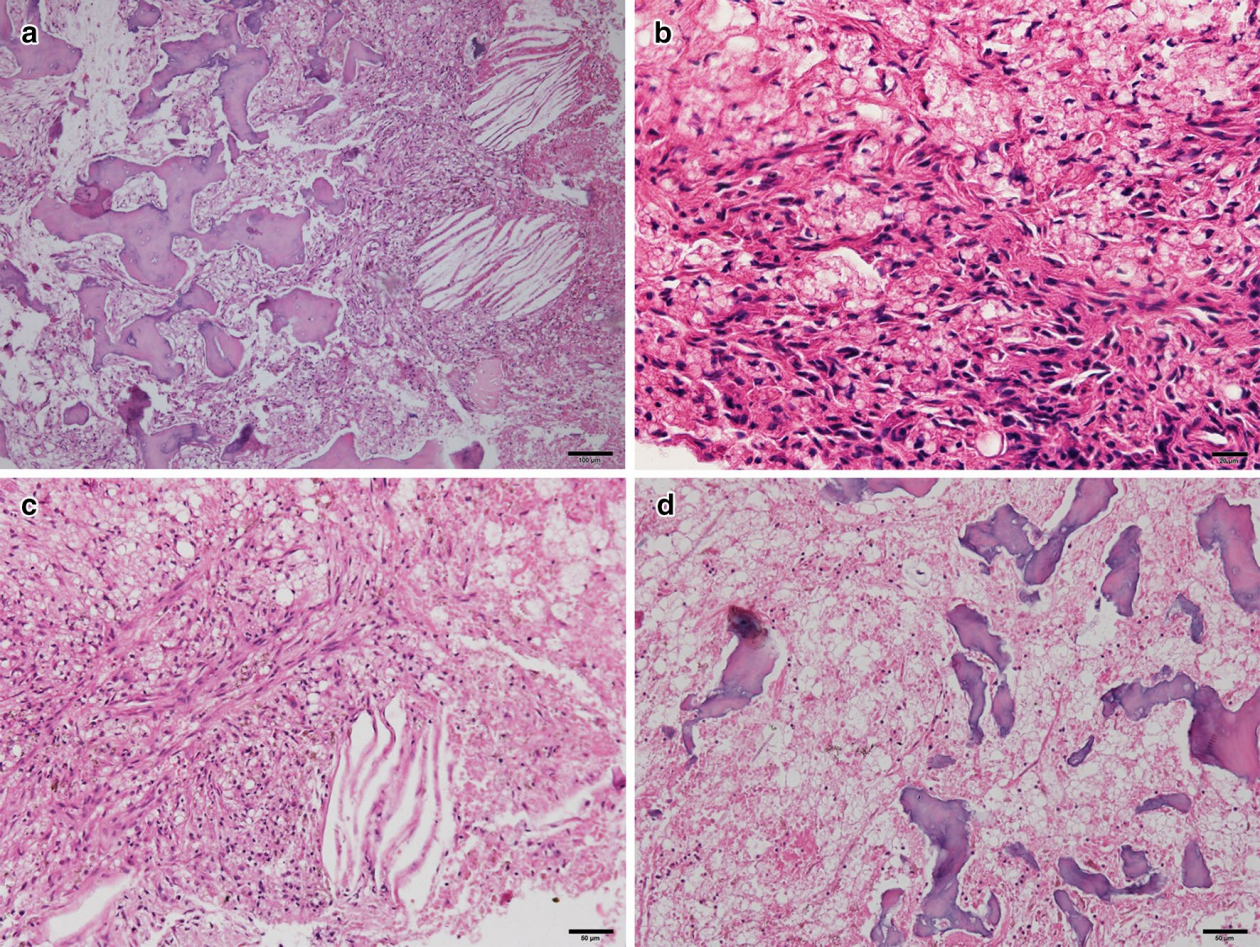
The hematoxylin and eosin staining view of the lesion. **a** Variable-sized woven bone was observed with a cholesterol cleft appearance under low magnification (*bar* 100 µm). **b** Under high magnification, the marrow stromal tissue was diffusely fibrosed and filled with fatty bubble-like materials, accompanied by the infiltration of many macrophages (*bar* 20 µm). **c** There also appeared to be several foci of the cholesterol slits (*bar* 50 µm). **d** The stromal fibrosis increased in the absence of vascular channels and advanced to the osteolytic trabecular bones, which showed irregular and rudimentary bony trabeculae with no signs of osteoid deposition (*bar* 50 µm)

An excisional biopsy of the left mandibular body was performed using the submandibular approach. Yellow colored semi-solid materials were removed from the bone marrow cavity (Fig. 2a). The lesion was poorly encapsulated, and the surrounding cortical bone had become thin but appeared to be intact (Fig. 2b). After removing the necrotic tissue, the wound was closed layer by layer. The removed specimen was sent for pathological examination. The removed specimen was fixed in 10 % neutral formalin and sectioned at a thickness of 4 μm. The microsections were routinely stained with hematoxylin and eosin (HE), and we also performed immunohistochemical (IHC) staining using anti-sera of lysozyme (DAKO, Glostrup, Denmark), matrix metalloprotease-1 (MMP-1, Santa Cruz Biotech, Santa Cruz, CA, USA), MMP-2 (Santa Cruz Biotech), MMP-3 (Santa Cruz Biotech), hypoxia inducible protein alpha (HIFα; Abcam, Cambridge, UK), vascular endothelial cell growth factor (VEGF; Abcam), osteoprotegerin (OPG; Santa Cruz Biotech), and the receptor activator of nuclear factor-kappa B ligand (RANKL; Santa Cruz Biotech). IHC staining was performed using the indirect triple sandwich method. The use of the present biopsy specimen was filed in the Department of Oral Pathology, Gangneung-Wonju National University Dental Hospital, and was approved by our institutional review board (IRB2015-7). The sections of the embedded specimens were stained with HE. The specimens contained inflammatory cells and fibroblastic cells. Variable sized woven bone was observed with a cholesterol cleft appearance (Fig. 3a). The marrow stromal tissue was diffusely fibrosed and filled with fatty bubble-like materials and was infiltrated with a large number of macrophages (Fig. 3b). Most of the adipocytes were destroyed and disappeared, resulting in the replacement of the fatty necrosis with variable sized vacuolated empty spaces. There also appeared to be several foci of the cholesterol slits, where fatty materials were also removed during the histological procedures (Fig. 3c). Stromal fibrosis increased in areas lacking vascular channels and advanced to the osteolytic trabecular bones, which showed irregular and rudimentary bony trabeculae with no signs of osteoid deposition and bisphosphonate-related multiple reversal lines (Fig. 3d).

**Fig. 4:**
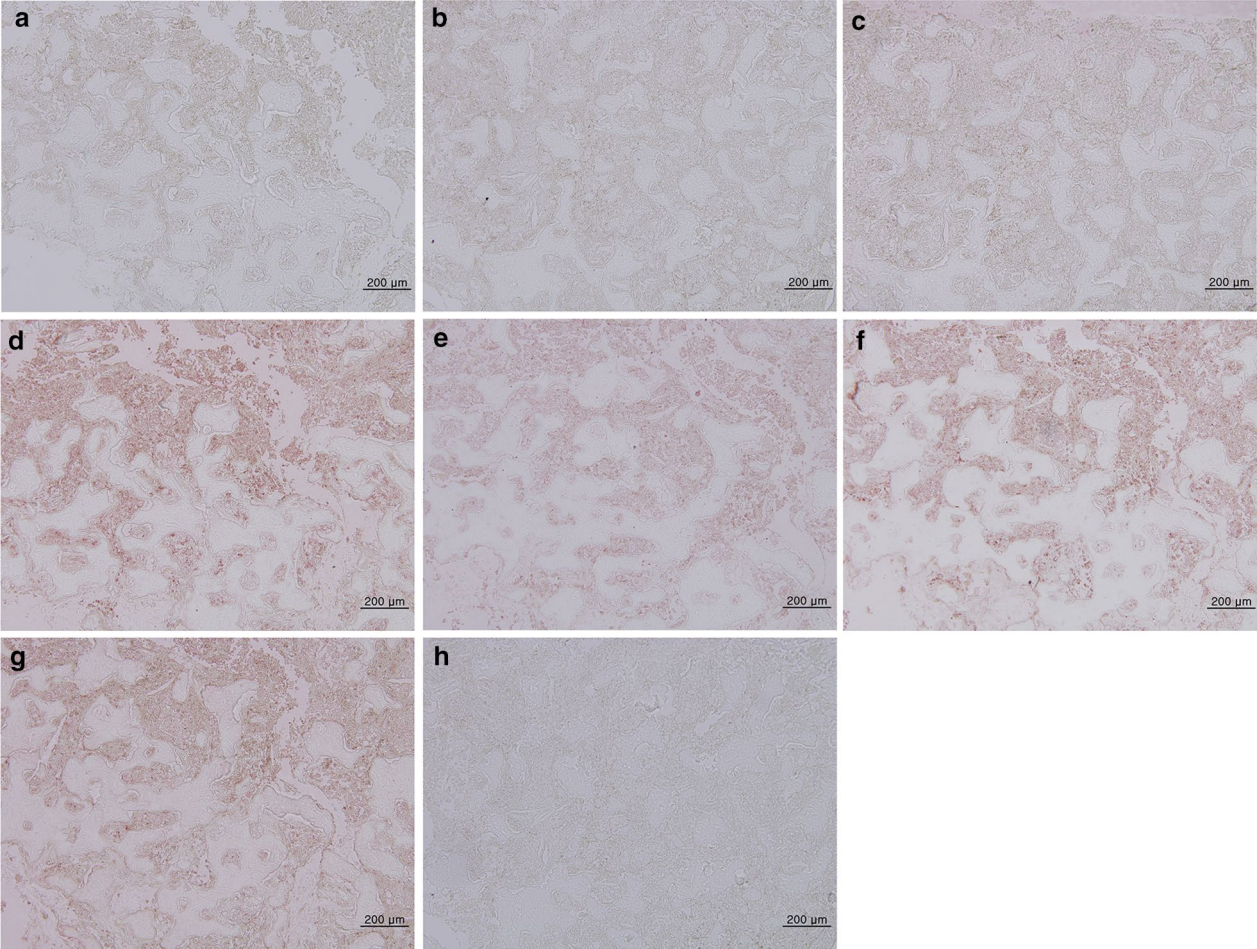
An immunohistochemistry view of the lesion without counterstain. **a** Matrix metalloproteinase (MMP)-1. **b** MMP-2. **c** MMP-3. **d** Lysozyme. **e** Receptor activator of nuclear factor-kappaB ligand (RANKL). **f** Osteoprotegerin (OPG). **g** Hypoxia inducible protein alpha (HIFα). **h** Vascular endothelial growth factor (VEGF)

In the immunohistochemistry analysis, the marrow stromal tissue was weakly positive for MMPs and the infiltrated macrophages were strongly positive for lysozymes (Fig. 4). Some marrow cells near the osteolytic trabecular bones were strongly positive for RANKL and OPG, and the stromal fibrous tissue was consistently positive for HIFα, but rarely positive for VEGF.

**Fig. 5:**
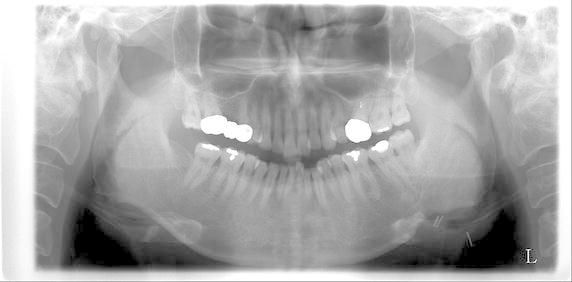
Postoperative panoramic radiography. The bone healing was evident in the left mandibular notch area at 5 months postoperatively

After surgery, the patient was advised to stop taking Synatura^®^ and olmesartan, which are anti-angiogenic drugs. The patient’s symptoms disappeared during follow-up and bone healing was uneventful at 5 months postoperatively (Fig. 5). The patient was followed up for 9 months postoperatively. No event occurred during follow-up.

## Discussion

Medication-related osteonecrosis of the jaw is frequently observed in the jaw bone (Khosla et al. 2007; Ruggiero et al. 2009). The drugs known to most commonly induce osteonecrosis are bisphosphonate (Abd-Alhaseeb et al. 2014; Balli et al. 2014) and denosumab (Sivolella et al. 2013). According to the patient’s medication history, the prescription period of anti-hypertension and anti-hyperlipidemia drugs was less than 2 months. Therefore, both drugs may have potentiated the progress of the osteonecrosis, but did not induce osteonecrosis in this patient. As the patient has pneumoconiosis, the bone loss of the mandible might be due to the patient’s systemic disease. As the severity of pneumoconiosis increases, the occurrence of bone loss is significantly higher (Li et al. 2012). However, other bones did not display osteoporosis. In addition, pneumoconiosis-related jawbone necrosis has not been reported before. Thus, the observed jaw bone necrosis might be due to the medications that were used.

The patient’s recent use of atorvastatin increased VEGF expression in the periodontium (Balli et al. 2014). Four different types of drugs had been prescribed to the patient over 3 years. Among them, we could not find any reference suggesting that ozagrel, erdostein, or acebrophyline might induce osteonecrosis or anti-angiogenesis. Synatura^®^is produced by a Korean pharmacologic company and is a type of herbal medicine. Synatura^®^ is a mixture of ivy leaf extract and coptis stalk extract. One active component of the coptis stalk is berberine (Min et al. 2006). Berberine has anti-angiogenic activity via suppression of VEGF expression (Hamsa and Kuttan 2012; Jie et al. 2011). Drugs with anti-angiogenic activity, such as bisphosphonates, may induce osteonecrosis of the jaw bone (Khosla et al. 2007; Ruggiero et al. 2009; Sivolella et al. 2013). In addition, the olmesartan that the patient took from February 2015 also has an anti-angiogenic effect (Abd-Alhaseeb et al. 2014). Accordingly, the observed osteonecrosis of the jaw might be caused by the combined effect of multiple drugs.

Contrast to the bisphosphonate-related osteonecrosis of the jaw, the present case showed that the osteolytic trabecular bones were scattered in the periphery of the main lesion and became rudimentay in shape (Fig. 3). In addition, osteolytic lesion was not associated with dental lesion (Fig. 1). Typical MRONJ has been associated with dental lesion or dental procedure (Katsarelis et al. 2015; Chandra et al. 2009). Most of osteocytes in the lacuna spaces were still vivid, rarely resulted in bony sequenstrum (Fig. 3). The marrow stromal tissue had extremely low vascularity and diffuse fatty necrosis, resulting in the formation of many vacuolated empty spaces and cholesterol slits (Fig. 3). The infiltrated macrophages were strongly positive for lysozymes, but did not produce an exudative inflammatory reaction (Fig. 4). Unlike typical MRONJ, as the necrotic jaw bone was not associated with bacterial infection, a severe inflammatory reaction was not observed. The stromal fibrous tissue was gradually degenerated with low expressions of MMPs (Fig. 4), but showed no evidence of reparative granulation tissue (Fig. 3). Therefore, we hypothesized that the stromal fibrous tissue was sclerosed retrogressively and lost its osteogenic properties. However, some stromal cells near the resorbing trabecular bones showed strong immunoreactions to RANKL and OPG (Fig. 4). This finding might indicate that the bone marrow was continuously modified by RANKL osteoclastic activity, but was not adequately replaced by OPG osteogenic activity due to the severe degeneration of the stromal fibroblasts. These findings demonstrate that the presented osteonecrosis underwent a chronic and persistent granulomatous inflammatory reaction (Jang et al. 2015). Additionally, the strong positive reaction of HIFα might indicate that the marrow stromal tissue was in a hypoxic condition, but this condition was not accompanied by de novo angiogenesis because of the sparse VEGF expression (Fig. 4).

Most of reported MRONJ cases have a history of dental procedure and spontaneous MRONJ cases also have been rarely reported (Chandra et al. 2009). Presented case was also developed MRONJ spontaneously. Though bisphosphonate has been mostly known to induce MRONJ, other inhibitors of angiogenesis can be implicated in isolated MRONJ (Pakosch et al. 2013). When MRONJ is involved the inferior alveolar nerve, the lesion may cause a painful neuropathy (Zadik et al. 2012; Kim et al. 2016). The lesion of our case was also involved the inferior alveolar nerve, causing pain. The limitation of our study was that 9 months of follow-up was not enough to demonstrate the relation between drug holiday and resolution of the lesion. A follow-up of 1–2 years of the present case without recurrence and further drug holiday might be needed.

## Conclusions

We conclude that the present case might have been caused by anti-angiogenic drug abuse, affecting the reduction of the mandibular marrow vascularity and subsequently inducing fatty necrosis and an extensive osteolytic change of the mandible. The localized lesion was found in the mandibular body without any dental lesion or bacterial infection, which are not typical of ordinary MRONJ.

## Patient consent

The patient has given his consent for the use of his personal and medical information for this case report.

## Abbreviations

MRONJ: medication-related osteonecrosis of the jaw
IHC: immunohistochemical
MMP-1: matrixmetalloprotease-1
MMP-2: matrix metalloprotease-2
MMP-3: matrix metalloprotease-3
VEGF: vascular endothelial cell growth factor
OPGO: steoprotegerin
RANKL: the receptor activator of nuclear factor-kappa B ligand
